# Internal carotid artery stenosis: hemodynamics in the ipsilateral ACA affects CT angiography manifestations

**DOI:** 10.3389/fnins.2023.1129570

**Published:** 2023-05-18

**Authors:** Chun Li, Jihong Meng, Baoxin Xu, Xiaodong Li, Miaomiao Li, Xue Du, Shaoyi Li, Weining Ma

**Affiliations:** ^1^Department of Pediatrics, Shengjing Hospital of China Medical University, Shenyang, China; ^2^Department of Neurosurgery, Shengjing Hospital of China Medical University, Shenyang, China; ^3^Department of Neurology, Shengjing Hospital of China Medical University, Shenyang, China

**Keywords:** anterior cerebral artery a1 segment, CT angiography, internal carotid artery stenosis, transcranial Doppler, hemodynamics

## Abstract

**Objective:**

This study aimed to evaluate whether CT angiography (CTA) manifestations in anterior cerebral artery a1 segment (A1) were related to the hemodynamics in patients with internal carotid artery stenosis (ICAS).

**Methods:**

A total of 97 cases were selected. The degree of ICAS and symmetry of A1 were evaluated by CTA examination. Hemodynamic indexes were detected by transcranial Doppler (TCD). The differences in CTA presentations of A1 and hemodynamics between the vessels on the stenotic and contralateral sides were analyzed according to the different degrees of stenosis. The degree of ICAS according to the different manifestations of A1 and the hemodynamics of A1’s adjacent vessels were also analyzed.

**Results:**

In the case of unilateral ICAS, the difference in Vm of A1 between the stenotic and the contralateral side was the most significant relative to the stenosis degree. When unilateral ICAS was ≥70%, the presentation of A1 on the stenotic side was more slender or non-visualized compared to that on the contralateral side, while in cases with unilateral stenosis <70% or bilateral stenosis with a similar degree of stenosis, A1 were mainly symmetrical. When A1 on the side of ICAS was slender or non-visualized, the Vm of A1 was significantly slower than that on the contralateral side (*P* < 0.001).

**Conclusion:**

The CTA manifestations of A1 on the side of ICAS embodied the overall changes of the intracranial hemodynamics after ICAS. A combination of TCD and CTA examination of A1 can assist in judging the location and degree of ICAS.

## Introduction

In our clinical practice, asymmetry of the anterior cerebral artery a1 segment (A1) bilaterally was commonly found in CT angiography (CTA) examination in patients with internal carotid artery stenosis (ICAS). The circle of Willis is an important channel for the compensation of intracranial blood flow in ICAS (Hartkamp et al., 1999). Previous studies have shown many possible variations in the circle of Willis, with an abnormal development of the A1 accounting for 6.7% of these (Krabbe-Hartkamp et al., 1998). A variation in the circle of Willis is more common in patients that have suffered a cerebrovascular accident (CVA) (De Caro et al., 2021), and the abnormal development of A1 may be a risk factor for ischemic symptoms in patients with ICAS (Van Seeters et al., 2015). It is well known that ICAS is a common neurological disease that accounts for approximately 10% of CVAs (Abbott et al., 2007). Owing to intracranial blood flow self-regulation, the ipsilateral hypoperfusion in cases of unilateral ICAS causes a change in the intracranial hemodynamics that facilitates collateral circulation (Henderson et al., 2000). The shunt from the anterior communicating artery (AcoA) to the A1 of the anterior cerebral artery (ACA) is a crucial primary channel of collateral circulation. Based on the experience of previous studies, we might attribute the asymmetry of the A1 branch bilaterally in ICAS to normal variation; however, according to the anatomy of the Circle of Willis, A1 may be the hemodynamic intersection of primary and secondary collateral circulation compensation after unilateral ICAS. Therefore, we ask the question of whether the imaging findings of A1 are related to the hemodynamics of this region during ICAS? To answer this, this study used a transcranial Doppler (TCD) to detect the hemodynamics of major intracranial and extracranial vessels, including adjacent vessels around the A1, and compared them with the CTA examination. To provide a clinical basis for the judgment of the degree of ICAS and treatment selection, a classification was made according to the degree of ICAS, and the relationship between hemodynamics and imaging of adjacent vessels around A1 was explored.

## Materials and methods

### Case selection

Cases were selected from the Department of Neurosurgery, Shengjing Hospital of China Medical University. The degree of ICAS and the imaging manifestations of main intracranial and extracranial vessels were determined by CTA examination. The stenosis site of internal carotid artery (ICA) was located at the bifurcation of common carotid artery (CCA) in enrolled cases. The degree of ICAS was calculated according to the North American Symptomatic Carotid Endarterectomy Trial (NASCET) Steering Committee (1991) criteria. Cases with unilateral ICAS that had an ipsilateral stenosis degree ≥50% with a contralateral ICAS degree <50% and bilateral stenosis (cases with bilateral ICAS ≥50%) were included, where a difference in the degree of stenosis ≥10% was defined as a severe stenosis (on the worse side), and <10% was defined as similar bilateral stenosis. Cases with intracranial vasogenic diseases, such as moyamoya disease, vascular malformation, and intracranial aneurysm, were excluded. A total of 97 cases of ICAS between June 2019 and June 2021 met the criteria, which included 75 unilateral and 22 bilateral ICAS cases. This study was approved by the Ethics Committee of Shengjing Hospital of China Medical University.

### Transcranial Doppler detection methods

All enrolled patients underwent TCD for the detection of major intracranial and extracranial vessels that included the CCA, ICA, middle cerebral artery (MCA), A1, posterior cerebral artery (PCA), ophthalmic artery (OA), supratrochlear artery (StrA) and siphonage of internal carotid artery (CS). A digital dual-channel, multi-depth TCD instrument and pulse Doppler probe (frequency 1.6 and 4.0 Hz; MVU6203, Delica, China) were used. Bilateral MCAs, A1 branches and PCAs were detected in the supine temporal window, and bilateral OAs and CSs were detected in the orbital window (detection depth: MCA, 40–75 mm; A1 branch, 60–75 mm; PCA, 55–75 mm; OA, 40–50 mm; CS, 40–75 mm). Bilateral CCAs, proximal ICAs, and StrAs (at the inner canthus) were detected in the supine position with a 4.0 Hz probe. In addition, cervical compression was used to determine the opening of the ipsilateral posterior communicating artery (PcoA). The blood flow direction, systolic and end diastolic blood flow velocity were measured, and the mean blood flow velocity (Vm) and pulse index (PI) were calculated (blood flow direction: toward the probe, CCA, MCA, PCA, OA, StrA and CS; deviate away from probe: ICA and A1).

### Classification of A1 imaging findings

The A1 was classified into three categories according to the imaging characteristics of bilateral A1 in CTA, namely: symmetrical A1; unilateral slender A1 (the diameter of the ipsilateral A1 is less than two-thirds of that of the contralateral one); and unilateral A1 that was not visualized on imaging.

### Research methods

The unilateral and bilateral stenosis cases were first grouped according to the degree of ICAS that was classified according to the CTA by NASCET criteria. Unilateral stenosis cases were classified into five groups according to the degree of stenosis that were 50–69%, 70–79%, 80–89%, 90%–near complete occlusion, and complete occlusion. According to the group, the Vm, blood flow direction, PI value, wall shear stress (WSS) of the A1, and imaging manifestations of A1 on both sides were recorded. Finally, based on the imaging manifestations of A1, the proportion of the degree of ICAS under different manifestations of A1, the Vm of A1 and MCA on both sides, blood flow direction and PI value were recorded.

### Statistical methods

When the patients were grouped according to the degree of ICAS and A1 performance, the age of the patients, Vm of each vessel and PI values were represented by the mean ± standard deviation. The paired sample *t*-test (two-tailed) statistical method was used to calculate the statistical differences in Vm and PI values between the ipsilateral stenosis vessels and the contralateral vessels. Multiple comparisons between groups were performed using the Bonferroni method. The Chi-square independence test was used to compare the differences in the case distribution of ICAS in different manifestations of A1. SPSS 26.0 software was used for statistical analysis, and statistical significance was considered with a *P* value < 0.05.

## Results

### Case characteristics

The average age of the 97 cases enrolled was of 64 ± 7 years, with 82 males and 15 females. Of the 75 unilateral ICAS cases, 15 were classified into in the 50–69% degree of stenosis group, 10 in the 70–79% group, 22 in the 80–89% group, 19 in the 90%–near complete occlusion group, and 9 in the complete occlusion group. Of the 22 bilateral ICAS cases, 16 were considered unilateral severe stenosis and six were similar bilateral stenosis (Table 1).

**Table 1:** Data of enrolled cases.

	Degree of stenosis	Number of cases	Age (years)	Gender	Symptoms
Unilateral ICAS	50–69%	15	64 ± 5	M: 12 F: 3	Asymptomatic: 9 TIA: 3 Cerebral infarction: 3
	70–79%	10	62 ± 7	M: 8 F: 2	Asymptomatic: 5 TIA: 2 Cerebral infarction: 3
	80–89%	22	65 ± 8	M: 15 F: 7	Asymptomatic: 7 TIA: 11 Cerebral infarction: 4
	90%-near complete occlusion	19	62 ± 8	M: 18 F: 1	Asymptomatic: 5 TIA: 12 Cerebral infarction: 2
	Complete occlusion	9	62 ± 8	M: 8 F: 1	Asymptomatic: 3 TIA: 3 Cerebral infarction: 3
Bilateral ICAS	More stenosis on one side	16	65 ± 6	M: 15 F: 1	Asymptomatic: 4 TIA: 5 Cerebral infarction: 1
	Similar bilateral stenosis	6	68 ± 7	M: 6 F: 0	Asymptomatic: 2 TIA: 1 Cerebral infarction: 3

TIA, transient ischemic attack.

### Hemodynamics of bilateral A1

**Figure 1:**
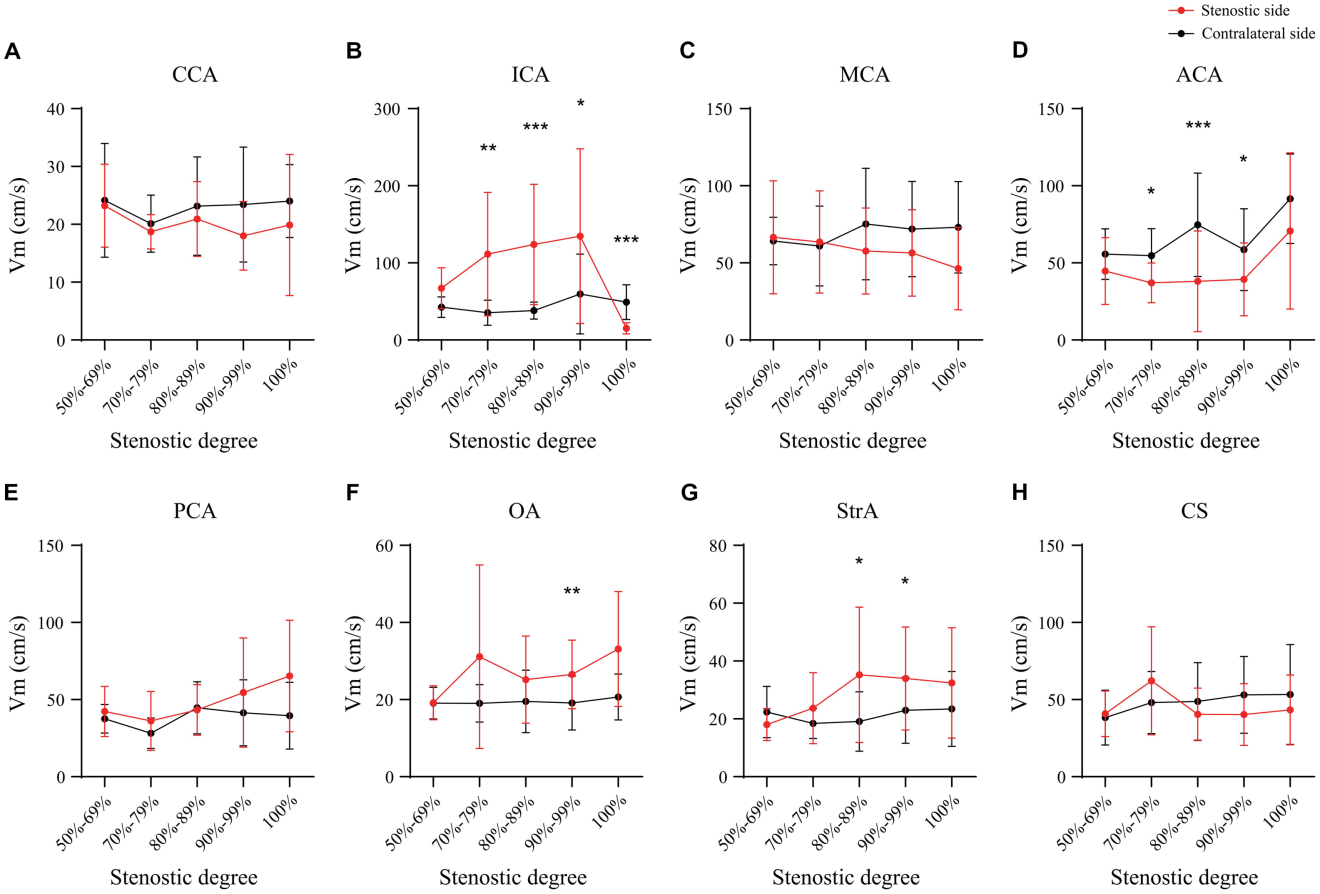
Mean cerebral blood flow velocity of main intracranial and extracranial vessels in unilateral ICAS. CCA, common carotid artery; ICA, internal carotid artery; MCA, middle cerebral artery; ACA, anterior cerebral artery; PCA, posterior cerebral artery; OA, ophthalmic artery; StrA, superior trochlear artery; CS, siphonage of internal carotid artery; ICAS, internal carotid artery stenosis; *p < 0.05; **p < 0.01; ***p < 0.001.

**Figure 2:**
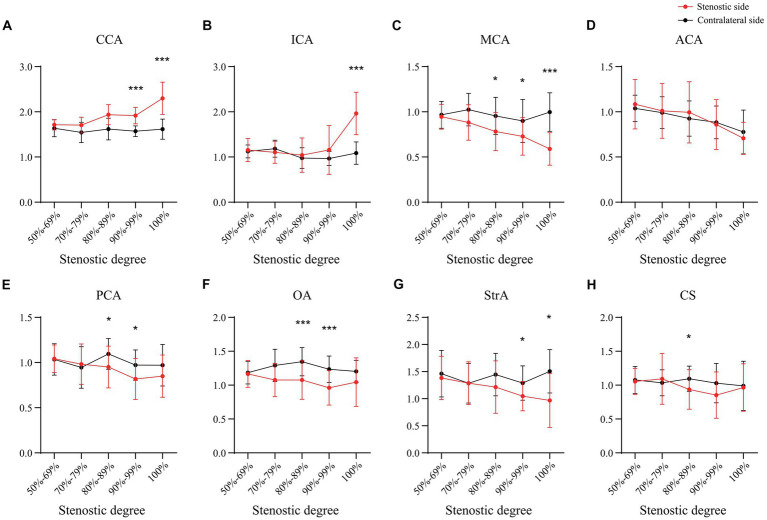
The pulse index of main intracranial and extracranial vessels in unilateral ICAS. CCA, common carotid artery; ICA, internal carotid artery; MCA, middle cerebral artery; ACA, anterior cerebral artery; PCA, posterior cerebral artery; OA, ophthalmic artery; StrA, superior trochlear artery; CS, siphonage of internal carotid artery; ICAS, internal carotid artery stenosis; *p < 0.05; **p < 0.01; ***p < 0.001.

**Figure 3:**
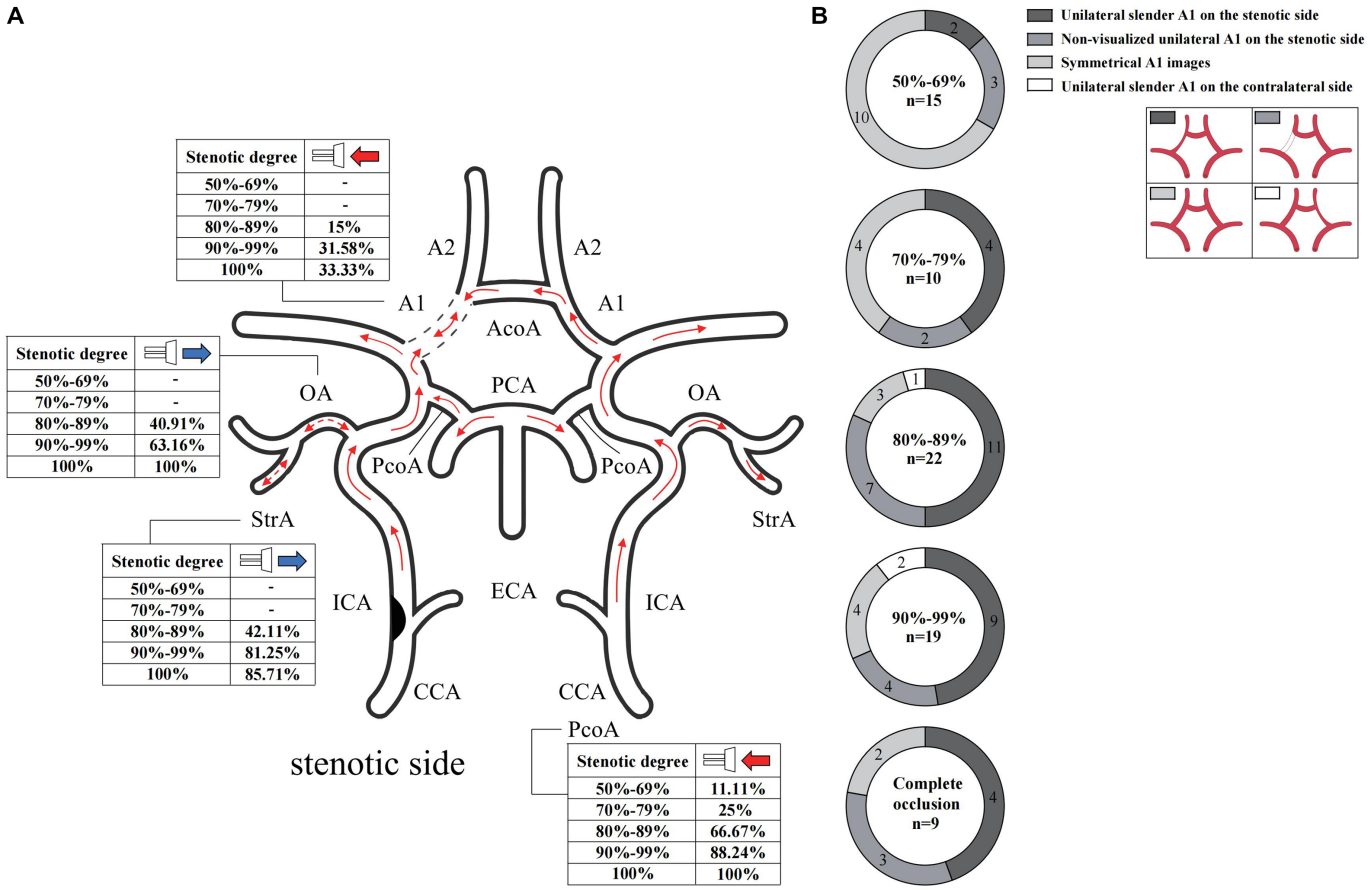
Blood flow direction and CTA manifestation of A1 in unilateral ICAS. **(A)** Blood flow direction. **(B)** CTA manifestation of A1. The numbers in the pie chart within the figures represent the number of cases. CTA, commuted tomography angiography; ICAS, internal carotid artery stenosis.

In the case of the unilateral ICAS, difference in hemodynamics of the ICA bilaterally was obviously related to the improvement of the degree of stenosis. In intracranial vessels, the hemodynamic difference of A1 bilaterally, that initially increased but then decreased with the increased degree of ICAS, was observed to be the most statistically significant (Figures 1, 2). When the degree of stenosis was 50–69%, there was no statistically significant difference observed in the Vm of A1 bilaterally (*P* > 0.05). When the degree of stenosis was 70–79%, the Vm of the A1 on the stenotic side was lower than that of the contralateral side (*P* < 0.05). When the degree of stenosis was 80–89%, the difference observed in the Vm of A1 bilaterally was the most obvious (*P* < 0.001), and the direction of blood flow was toward the probe in 15% of these patients. When the degree of stenosis was 90% to near complete occlusion, the difference observed in the Vm of A1 bilaterally decreased (*P* < 0.05). In 31.58% of these cases, the direction of blood flow in the A1 on the stenotic side was toward the probe. When an ICA was completely occluded, there was no statistical difference observed in the Vm of A1 bilaterally (*P* > 0.05), and the direction of blood flow in the A1 of the stenotic side was toward the probe in 33.33% of these cases. It should be noted that when the degree of internal carotid artery stenosis reached over 80%, the PI value of the stenotic MCA decreased when compared with that of the contralateral side, and the opening of the OA and StrA secondary collateral circulation gradually increased. When the ipsilateral internal carotid artery was completely occluded, the OA on the stenotic side of all enrolled cases showed a reverse direction of blood flow. In addition, we found that as the degree of stenosis increased, the proportion of ipsilateral PcoA opened and supplied blood to the anterior circulation increased gradually (Figure 3A). When multiple comparisons were made between groups according to the degree of stenosis, the significance level of the mean difference of A1 was obvious (Table 2).

**Table 2:** Multiple comparisons between groups by degree of stenosis.

Index	Side	Vessel	Significance (between groups)	Multiple comparison (Bonferroni)
Vm	Stenotic side	ICA	0.002	0.08 (3 vs. 5)
				0.03 (4 vs. 5)
		MCA	0.584	
		A1	0.068	
		OA	0.076	
		StrA	0.087	
	Contralateral side	ICA	0.14	
		MCA	0.666	
		A1	0.004	0.01 (1 vs. 5)
				0.046 (2 vs. 5)
		OA	0.987	
		StrA	0.739	
PI	Stenotic side	ICA	0	0.000 (1 vs. 5)
				0.000 (2 vs. 5)
				0.000 (3 vs. 5)
				0.000 (4 vs. 5)
		MCA	0	0.017 (1 vs. 4)
				0.000 (1 vs. 5)
				0.014 (2 vs. 5)
		A1	0.023	0.032 (1 vs. 5)
		OA	0.291	
		StrA	0.147	
	Contralateral side	ICA	0.012	0.044 (1 vs. 4)
		MCA	0.566	
		A1	0.018	0.017 (1 vs. 4)
		OA	0.145	
		StrA	0.573	

Five groups according to the degree of stenosis. 1: 50–69%; 2: 70–79%; 3: 80–89%; 4: 90%–near complete occlusion; 5: complete occlusion.

**Figure 4:**
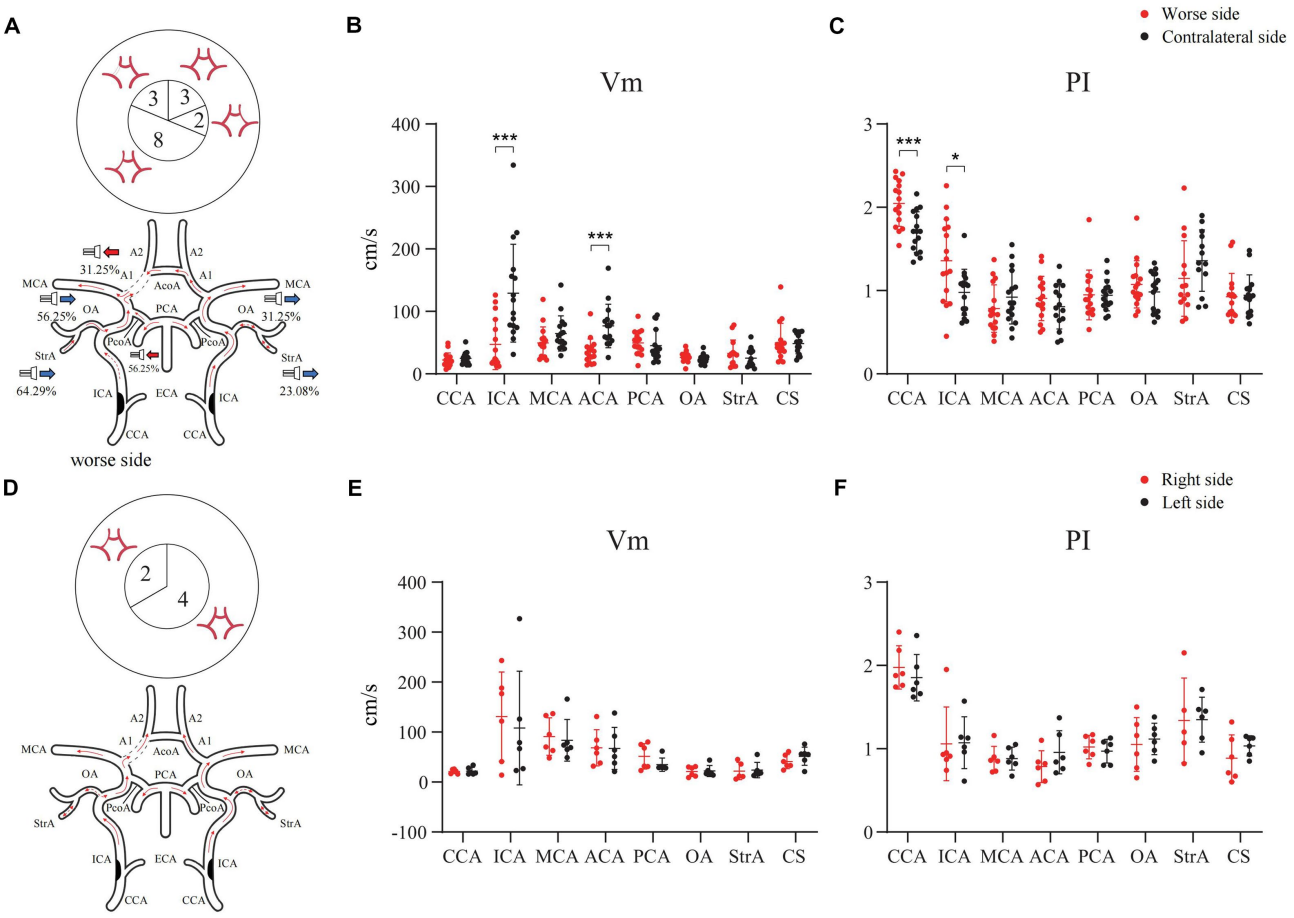
Hemodynamics of main intracranial and extracranial vessels and CTA expressions of A1 in bilateral ICAS. **(A)** Severe stenosis of one ICA. **(B)** The difference in mean velocity of blood flow between bilateral vessels in severe stenosis of one ICA. **(C)** The difference in pulse index between bilateral vessels in severe stenosis of one ICA. **(D)** The degree of stenosis of the bilateral ICAs is similar. **(E)** The difference in mean velocity of blood flow between bilateral vessels in patients with similar degree of bilateral stenosis. **(F)** The difference in pulse index between bilateral vessels in patients with similar degree of bilateral stenosis. The numbers in the pie chart within the figures represent the number of cases. CTA, commuted tomography angiography; ICA, internal carotid artery; ICAS, internal carotid artery stenosis; *p < 0.05; ***p < 0.001.

In the case of the bilateral ICAS, Vm in the A1 of the stenotic more severe side was significantly lower than that of the contralateral side (*P* < 0.001), except for the difference in the hemodynamics when the CCA and ICA were compared. In 31.25% of the cases, the direction of blood flow of the A1 on the stenotic side was toward the probe. In 56.25% of the cases, the PcoA on the worse side was open and supplied to the ipsilateral anterior circulation. In addition, both the OA and StrA secondary collateral circulation showed varying degrees of opening in cases with both severe and relatively mild stenosis (Figures 4A–C). However, there was no statistically significant difference in the hemodynamics of bilateral intracranial and extracranial vessels in the group with similar degrees of bilateral stenosis (Figures 4D–F).

**Figure 5:**
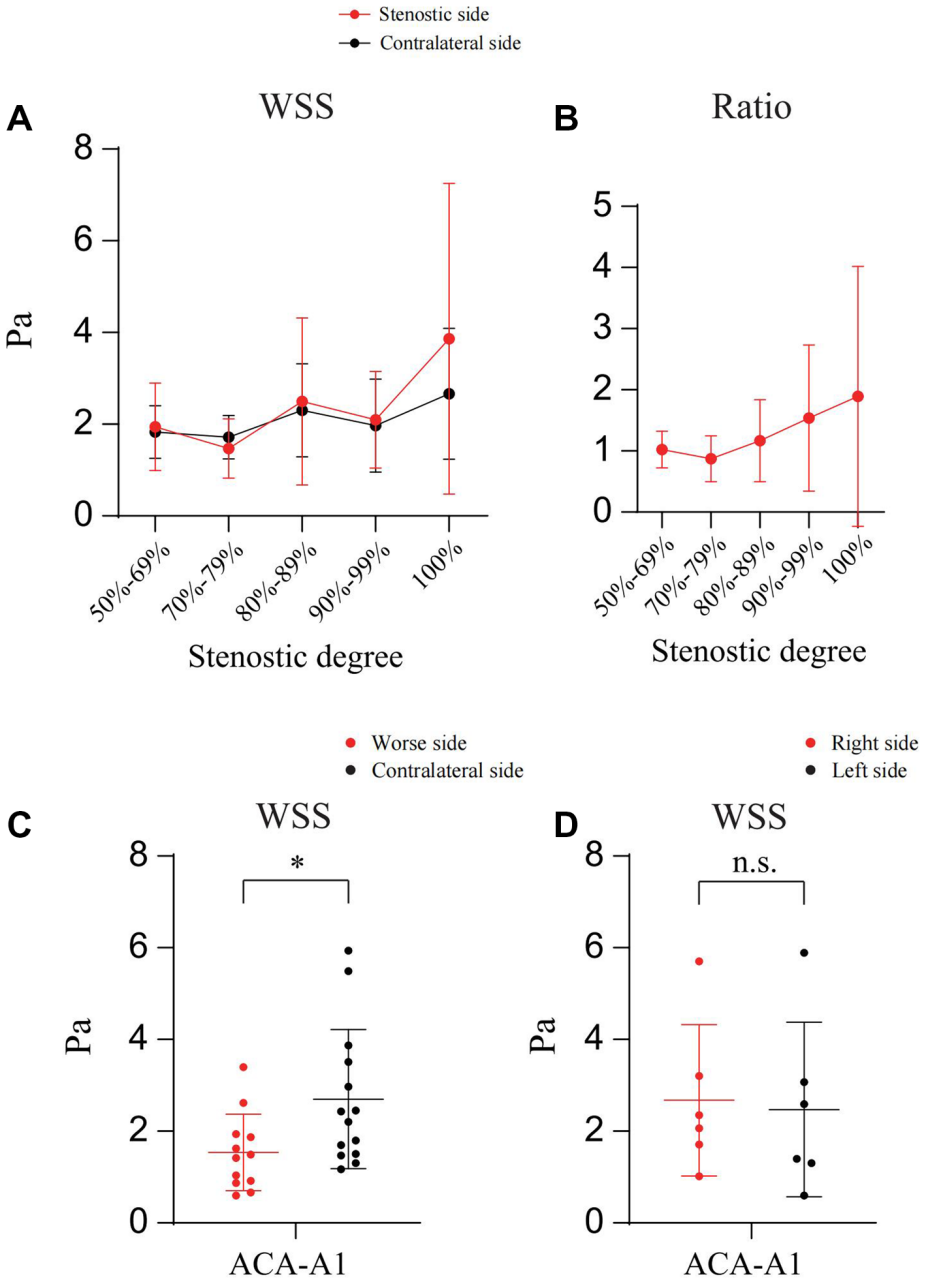
Calculation and comparison the WSS of A1 on both sides. **(A)** The WSS of bilateral A1 in patients with unilateral ICAS at different degrees. **(B)** WSS ratio of bilateral A1 in patients with unilateral ICAS at different degrees. **(C)** WSS of bilateral A1 in patients with bilateral ICAS (severe stenosis on one side). **(D)** WSS of bilateral A1 in patients with bilateral ICAS (similar degree of bilateral stenosis); *p < 0.05.

In addition, we calculated and compared the WSS (Poiseuille’s Law) (Malek et al., 1999) of A1 on both sides. As shown in Figures 5A,B, in cases of unilateral ICAS, although the ratio of WSS of A1 on the stenotic side to that of A1 on the contralateral side increased with the increase of stenosis degree, the difference was not statistically significant (*p* > 0.05). In cases of the bilateral ICAS, the WSS of A1 on the side with more severe stenosis was lower than that on the other side (*p* < 0.05) (Figure 5C), while in cases with similar bilateral stenosis, there was no significant difference in the WSS of A1 on both sides (*p* > 0.05) (Figure 5D).

### Unilateral slender and non-visualized A1 on the stenotic side

In cases of unilateral ICAS, the manifestations of A1 observed on CTA bilaterally varied according to the degree of ICAS. When the degree of stenosis was 50–69%, most A1 images were symmetrical. When the degree of stenosis was 70–79%, bilateral A1 symmetry observed on imaging decreased. When the degree of stenosis was in the range of 80–89%, majority of A1 on the stenotic side presented as slender or non-visualized. When the degree of stenosis was 90% to near complete occlusion, the proportion of A1 on the stenotic side that were slender or non-visualized was 68.42%. However, when the ICA was completely occluded, the proportion of occluded A1 that presented as slender or non-visualized was 77.78% (Figure 3B). In the bilateral ICAS cases, 68.75% of A1 on the severe stenotic side presented as slender or non-visualized (Figure 4A), while symmetric imaging was mainly observed in cases with similar degrees of bilateral stenosis (Figure 4D).

### Differences in hemodynamics of A1 adjacent vessels with different imaging manifestations

**Figure 6:**
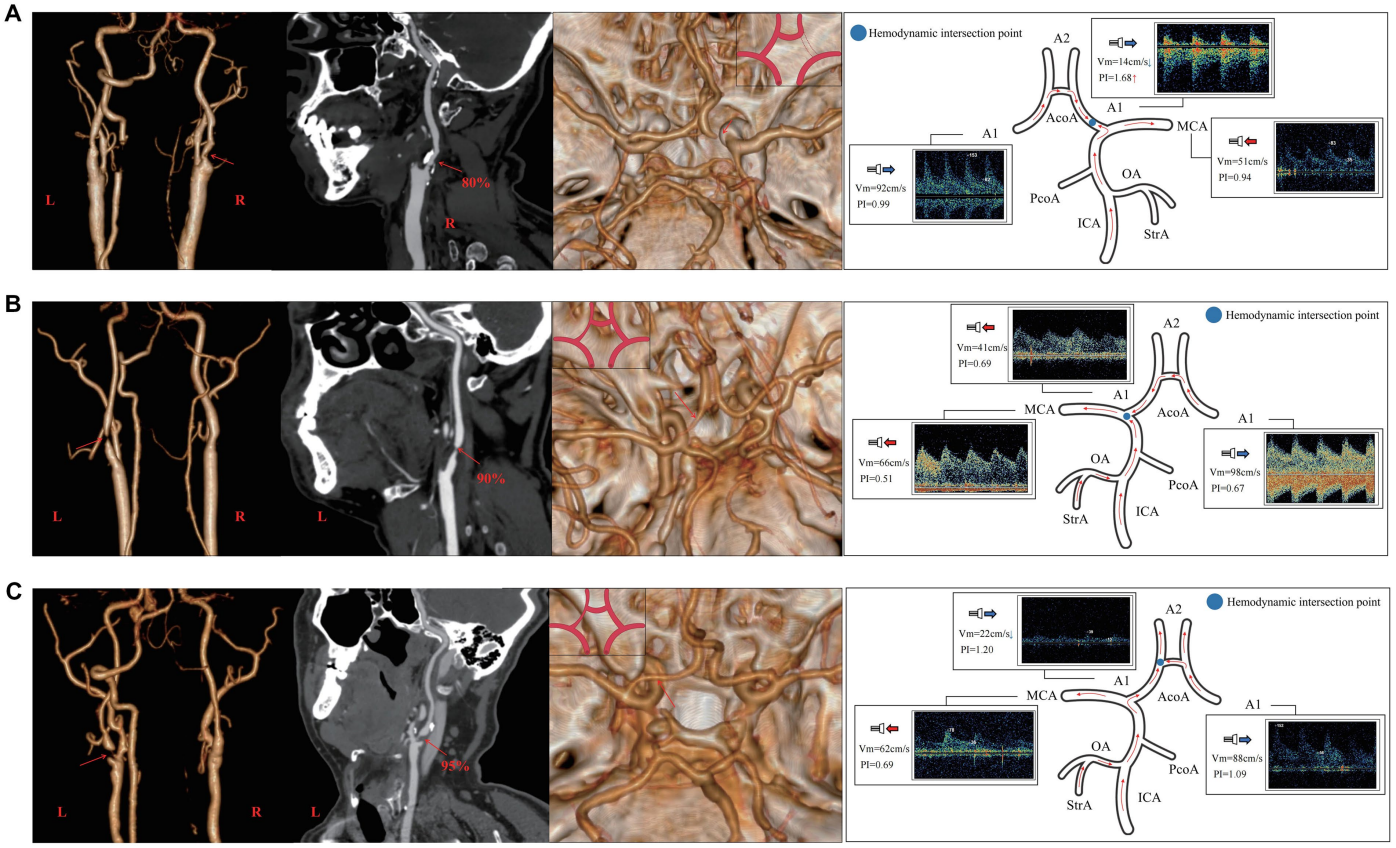
The relationship between the CTA manifestation of A1 on the side of stenosis and hemodynamics of the adjacent vessels of A1 in unilateral ICAS. **(A)** Case 1 has 80% stenosis in the right ICA. CTA shows that the right A1 was non-visualized, TCD suggests that the direction of blood flow in the right A1 deviated from the probe, Vm decreases, and PI value increases. **(B)** Case 2 presents with a 90% stenosis in the left ICA. CTA shows that A1 on the left was slender, while TCD suggests that the blood in A1 on the left flows toward the probe and its Vm decreases. **(C)** Case 3 has a 95% stenosis in the left ICA. CTA shows that the left A1 was relatively slender, TCD suggests that the blood flow of the left A1 deviates from the probe, and the Vm decreases. CTA, commuted tomography angiography; ICA, internal carotid artery; ICAS, internal carotid artery stenosis; TCD, transcranial Doppler; PI, pulse index; Vm, mean blood flow velocity.

As per the CTA manifestations of the unilateral A1 on the stenotic side, hemodynamic changes of A1 and MCA on both sides were observed. Among them, there were 22 cases of non-visualized unilateral A1 on the stenotic side, and seven cases (31.82%) had a degree of stenosis of 80–89%. In 17 of these cases, the direction of blood flow in the A1 on the stenotic side deviated from the probe (Figure 6A), and the difference in Vm between A1 on the stenotic and contralateral side was statistically significant (*P* < 0.001). There were a total of 38 cases of unilateral slender A1 on the stenotic side, among which 11 had a degree of stenosis of 80–89% (28.95%), and 9 had 90% to near complete occlusion (23.68%). In 10 of these cases, the direction of blood flow in the stenotic A1 was toward the probe (Figure 6B), while the other 28 cases deviated from the probe (Figure 6C). There was a statistically significant difference in Vm between the cases where the blood in the A1 on the stenotic and contralateral flowed in the direction away from the probe (*P* < 0.001). In addition, regardless of the direction of blood flow in the A1 on the stenotic side, there were statistically significant differences in the Vm and PI value for the MCA on both sides (*P* < 0.05). There were 26 cases with symmetrical A1 imaging, with the highest proportion of 10 cases in unilateral ICAS with a degree of stenosis of 50–69% (38.46%). In 21 cases, the direction of blood flow of the A1 on the stenotic side deviated away from the probe, and there was no statistically significant difference between the Vm and PI value of A1 and MCA bilaterally (*P* > 0.05; Table 3).

**Table 3:** The stenotic degree of ICA and the hemodynamics of A1’s adjacent vessels according to the CTA manifestations of A1 on the side of ICAS.

CTA manifestations of A1			Symmetrical		Slender		Non-visualized	
Cases numbers			26		38		22	
Stenotic degree of ICA			50–69%: 10 (38.46%) 70–79%: 4 (15.38%) 80–89%: 3 (11.54%) 90%-near complete occlusion: 4 (15.38%) Complete occlusion: 2 (7.69%) More stenosis on one side: 3 (11.54%)		50–69%: 2 (5.26%) 70–79%: 4 (10.53%) 80–89%: 11 (28.95%) 90%-near complete occlusion: 9 (23.68%) Complete occlusion: 4 (10.53%) More stenosis on one side: 8 (21.05%)		50–69%: 3 (13.64%) 70–79%: 2 (9.09%) 80–89%: 7 (31.82%) 90%-near complete occlusion: 4 (18.18%) Complete occlusion: 3 (13.64%) More stenosis on one side: 3 (13.64%)	
Flow direction of A1			− (*n* = 21)	+ (*n* = 5)	− (*n* = 28)	+ (*n* = 10)	− (*n* = 17)	+ (*n* = 5)
TCD	A1 on the stenotic side	Vm (cm/s)	48 ± 18	44 ± 8	38 ± 29	61 ± 51	30 ± 14	37 ± 32
		PI	0.96 ± 0.21	0.66 ± 0.17	1.04 ± 0.36	0.83 ± 0.19	1.03 ± 0.26	0.62 ± 0.20
	Contralateral A1	Vm (cm/s)	52 ± 19	92 ± 30	72 ± 34	71 ± 22	76 ± 34	75 ± 8
		PI	1.00 ± 0.21	0.82 ± 0.26	0.90 ± 0.22	0.84 ± 0.15	0.93 ± 0.18	0.84 ± 0.20
	*P* values		*P*v = 0.54 *P*p = 0.49	*P*v = 0.01 *P*p = 0.32	*P*v < 0.001 *P*p = 0.12	*P*v = 0.58 *P*p = 0.92	*P*v < 0.001 *P*p = 0.22	*P*v = 0.05 *P*p = 0.17
	MCA on the stenotic side	Vm (cm/s)	67 ± 32	57 ± 44	57 ± 29	45 ± 15	53 ± 22	55 ± 29
		PI	0.89 ± 0.16	0.63 ± 0.19	0.76 ± 0.23	0.60 ± 0.15	0.85 ± 0.22	0.75 ± 0.27
	Contralateral MCA	Vm (cm/s)	60 ± 22	47 ± 5	78 ± 33	67 ± 18	73 ± 27	69 ± 22
		PI	1.00 ± 0.27	1.07 ± 0.18	0.92 ± 0.22	0.96 ± 0.21	0.94 ± 0.16	0.88 ± 0.26
	*P* values		*P*v = 0.38 *P*p = 0.11	*P*v = 0.68 *P*v = 0.01	*P*v = 0.02 *P*p < 0.001	*P*v = 0.01 *P*p < 0.001	*P*v = 0.03 *P*p = 0.23	*P*v = 0.46 *P*p = 0.48

ICAS, internal carotid artery stenosis; MCA, middle cerebral artery; ACA, anterior cerebral artery; “+”: The blood flow direction toward the probe; “−”: The blood flow direction deviates away from the probe.

According to the symmetry or asymmetry (slender or non-visualized) of A1 on both sides, it can be seen that the degree of unilateral ICAS was usually more than 70% when A1 on the side of ICAS is slender or non-visualized (*P* < 0.01). The Vm in the A1 on the stenotic side was significantly lower than that on the contralateral side if the direction blood flow in A1 deviated from the probe (*P* < 0.001), regardless of whether the A1 on the stenotic side was slender or non-visualized.

## Discussion

CTA findings of A1 and overall changes of intracranial hemodynamics after ICAS.

Collateral circulation plays an important role in the pathophysiological process of cerebral ischemia (Liebeskind, 2003). It is particularly important to fully understand collateral circulation and to evaluate the treatment and prognosis of ischemic cerebrovascular disease in combination with hemodynamics (Hartkamp et al., 2018). After the occurrence of unilateral ICAS, the ipsilateral perfusion pressure will decrease accordingly, which may lead to adaptive changes *via* collateral circulation (Wang et al., 2019) and intracranial hemodynamics (Reinhard et al., 2003; Kamouchi et al., 2005). An example of this would be the opening of the primary collateral circulation represented by the circle of Willis, and the reverse flow compensated by the second collateral circulation represented by the OA, among others (Sekine et al., 2018). The ICA provides the blood supply for anterior cerebral circulation, and its main branches are the MCA and ACA. The A1 is found in the intersectional area of the blood supply from the ICA to the frontal lobe that diverts the blood from the contralateral ACA through the AcoA, which is the hemodynamic intersection point of the compensatory collateral circulation in the anterior circulation. Through the overall study of the hemodynamics of the main intracranial and extracranial vessels in cases of ICAS, we found that with the worsened severity of ICAS, the hemodynamics changed in the A1 ipsilateral to the stenosis and secondary collateral circulation represented by the OA and StrA. When the degree of unilateral ICAS was 70–79%, the Vm in A1 on the stenotic side decreased; when the degree of stenosis was 80–89%, the decrease in Vm in A1 was the most significant. It was at this point that the direction of blood flow of the ipsilateral OA and StrA reversed. When the degree of stenosis was >90%, there was no significant difference in the Vm in A1 on both sides. When the ICA was completely occluded, the direction of blood in the ipsilateral A1 tended to flow toward the probe, which indicates that the hemodynamics of A1 reflects on the overall intracranial hemodynamics after ICAS. Previous studies have shown that the total cerebral blood flow in patients with symptomatic carotid artery stenosis was redistributed, with the ACA as the main compensatory channel that can reflect the redistribution of blood flow bilaterally (Zarrinkoob et al., 2019), which is consistent with our results. Interestingly, when we further studied cerebral blood flow of patients with different degrees of stenosis on CTA, we observed that the A1 asymmetry increased as the degree of stenosis increased, which was significantly higher than that reported in previous studies (Krabbe-Hartkamp et al., 1998). In our study, regardless of the degree of ICAS, there was no statistically significant difference in the PI values of A1 bilaterally, which indicated the stenosis of the vessel itself could be excluded. Therefore, we speculated that when ICAS occurs, the slender or non-visualized phenomenon of the ipsilateral A1 on CTA was the overall embodiment of intracranial hemodynamics at A1 that can be understood as a “convection effect” of blood flow. Owing to the difference of the hemodynamics in A1 bilaterally, the difference in the retention time of the contrast agent led to the asymmetry observed on CTA. Specifically, when the total blood flow of the ipsilateral ICA, including the secondary collateral circulation, continued to supply the ipsilateral ACA, the convective point of cerebral blood flow was located at the intersection of the A2 segment at the distal end of A1 of the ipsilateral ACA and the AcoA. When the total blood flow of the ipsilateral ICA was the same as that from the shunt after the AcoA, the convective point of blood flow was in A1, with no effective blood flow observed in A1. When the ICA cannot provide additional blood flow to the ACA, the convective point of blood flow was at the bifurcation of the ipsilateral ACA and MCA, with the direction of blood flow in the A1 toward the probe. When the degree of stenosis in the ICA bilaterally was similar, both ICAs provided a similar blood flow to the intracranial vessels, and both A1 were mostly symmetrical on CTA; this indirectly confirmed our hypothesis. However, it should be noted that in some cases, the distal vessels of the ICA on the non-stenotic side had one or series stenosis. Therefore, if the degree of stenosis in one ICA is relatively high, both A1s can still be symmetrical. This indicates that the evaluation of intracranial hemodynamics for ICAS should be wholly analyzed.

### Identifying the degree and location of ICAS

Our study found that the hemodynamic changes in A1 on the side of ICAS were related to the degree of stenosis, and the CTA manifestations of A1 may be caused by its hemodynamics. Although CTA examination is an effective method to evaluate ICAS, in clinical practice (Osephson et al., 2004), cervical CTA or ultrasound examination is often not performed promptly in patients with ischemia, which may omit the consideration of the condition of neck vessels. Previous studies have shown that asymmetry of the intracranial segment of the ICA suggests severe stenosis in an ICA (Naggara et al., 2008). In cases of severe ICAS, a high proportion of unilateral A1 dysplasia or underdevelopment was observed on CTA that was attributed to the anatomic variation of the circle of Willis in previous studies (Waaijer et al., 2007; Varga et al., 2019). Although these results are consistent with our study, the hemodynamics of A1 have not yet been further studied. Through our TCD studies, it was found that when the A1 was slender or non-visualized on CTA, it was owing to the difference in hemodynamics of two sides of A1 caused by intracranial hemodynamic changes after ICAS rather than from stenosis or lack of blood flow. Therefore, clinicians should conduct a more comprehensive assessment of the CTA findings in combination with assessment of the hemodynamics when judging the pathogenesis of cerebral ischemia patients (Suwanwela et al., 2002; Tsivgoulis et al., 2007) to find the potential causes of ischemia, such as severe stenosis of ICA. In our study, the appearance of an A1 on CTA as slender or non-visualized, and a decreased Vm when compared with that on the contralateral side often predicted a severe ipsilateral ICAS (≥70%). Furthermore, it is suggested to also pay attention to the opening of the secondary collateral circulation, such as in the OA or StrA, for a more comprehensive judgment.

It is also important to note that the degree of stenosis in the ICA did not affect the Vm of the MCA on the stenotic side, which indicated that the hemodynamic changes caused by ICAS did not affect the distal vessels (Shakur et al., 2014, 2015). However, when the degree of ICAS was >80%, the PI value of the ipsilateral MCA decreased significantly when compared with that of the contralateral MCA; this suggested that when ipsilateral blood flow decreased due to ICAS, the terminal capillaries opened, vascular resistance decreased, and the PI value appeared to “dynamically decline.” Further research is needed to confirm this.

The limitation of this study is that there are no dynamic intracranial vascular changes, and longer observation and related hemodynamic mechanism research should be conducted in the future.

## Conclusion

Although variations in the circle of Willis are common, changes in hemodynamics may affect the imaging of intracranial vessels in ischemic cerebrovascular disease, especially at the location of blood flow convection. The symmetry of A1 is relative to hemodynamics rather than variations in the circle of Willis. In addition, the activation of the primary and secondary collateral circulation is relative to the degree of stenosis. The “convection effect” proposed in this study provides a basis for understanding the hemodynamic changes and pathological mechanisms associated with ICAS. Our study found that CTA imaging of A1 on the stenotic side after ICAS embodied the intracranial hemodynamics at A1. The combined findings of the hemodynamics of A1 bilaterally and the CTA examination can assist in judging the location and the degree of ICAS.

## Data availability statement

The original contributions presented in the study are included in the article/supplementary material, further inquiries can be directed to the corresponding authors.

## Ethics statement

The studies involving human participants were reviewed and approved by the Ethics Committee of Shengjing Hospital of China Medical University. The patients/participants provided their written informed consent to participate in this study.

## Author contributions

CL and WM: study concept and design, analysis and interpretation, and critical revision of the manuscript for important intellectual content. BX, ML, XL, and XD: acquisition of data, analysis, and interpretation. JM and SL: study concept and design, critical revision of the manuscript for important intellectual content, and study supervision. All authors also confirm accountability for the accuracy and integrity of the work.

## Conflict of interest

The authors declare that the research was conducted in the absence of any commercial or financial relationships that could be construed as a potential conflict of interest.

## Publisher’s note

All claims expressed in this article are solely those of the authors and do not necessarily represent those of their affiliated organizations, or those of the publisher, the editors and the reviewers. Any product that may be evaluated in this article, or claim that may be made by its manufacturer, is not guaranteed or endorsed by the publisher.

## Figures and Tables

**Table:** 

A1	anterior cerebral artery a1 segment
ACA	anterior cerebral artery
CCA	common carotid artery
CS	siphonage of internal carotid artery
CTA	CT angiography
CVA	cerebrovascular accident
ICA	internal carotid artery
ICAS	internal carotid artery stenosis
MCA	middle cerebral artery
NASCET	North American Symptomatic Carotid Endarterectomy Trial
OA	ophthalmic artery
PCA	posterior cerebral artery
StrA	supratrochlear artery
TCD	transcranial Doppler
TIA	transient ischemic attack
PI	pulse index
